# Intravitreal luxated lens stuck on the optic disc: a case report

**DOI:** 10.1186/s13256-016-0807-9

**Published:** 2016-01-22

**Authors:** Seita Morishita, Masanori Fukumoto, Hiroyuki Suzuki, Takaki Sato, Teruyo Kida, Eisuke Isizaki, Mari Ueki, Tetsuya Sugiyama, Tsunehiko Ikeda

**Affiliations:** 1 Department of Ophthalmology, Osaka Medical College, 2-7 Daigaku-machi, Takatsuki-City, Osaka 569-8686 Japan; 2 Department of Ophthalmology, Takatsuki Red Cross Hospital, Takatsuki-City, Osaka Japan; 3 Isizaki Eye Clinic, Nara, Japan; 4 Nakano Eye Clinic, Kyoto, Japan

**Keywords:** Lens luxation, Optic disc, Vitreous surgery, Residual vitreous gel

## Abstract

**Background:**

We encountered a rare patient with lens luxation in which the lens had become stuck on the optic disc. Findings obtained during vitreous surgery suggested that the luxated lens had become stuck on the optic disc via residual vitreous gel.

**Case presentation:**

An 88-year-old Japanese man experienced lens luxation into the inferior vitreous cavity while undergoing treatment for glaucoma in his left eye. Because no inflammation was present upon examination, we observed the patient without prescribing any additional medications except for the eye drop treatment for glaucoma. Two years later, the patient revisited our clinic after suddenly noticing a visual disturbance in his left eye. A fundus examination revealed that the luxated lens had become stuck on the optic disc and displayed no changes in relation to the patient’s head position or eye movement. Subsequently, vitreous surgery was performed to remove the luxated lens. During the surgery, we observed an aggregation of vitreous gel between the luxated lens and the optic disc. The luxated lens was successfully mobilized by pushing with a vitreous cutter and then extracted through a corneoscleral incision using perfluorocarbon liquid. At 4 months after surgery, the patient’s visual acuity had improved to 20/25.

**Conclusions:**

The findings of this study suggest that the luxated lens had become stuck on the optic disc via residual vitreous gel on the optic disc. The surgical procedure of extracting the luxated lens through a corneoscleral incision using perfluorocarbon liquid was found to be both safe and effective.

## Background

In patients with lens luxation into the vitreous cavity, lensectomy (intracapsular lens extraction or intravitreal phacoemulsification), vitrectomy, and intraocular lens ciliary sulcus fixation are often performed as treatment [1–4]. However, some patients who show no complications, such as decreased visual acuity (VA) or lens-induced endophthalmitis, are followed conservatively [5]. A luxated lens is usually located in the inferior vitreous cavity without adherence to the retina in the posterior pole [6]. In this report, we describe a rare case in which the luxated lens in the vitreous cavity became stuck on the optic disc and obstructed the macular region, thus causing a marked visual disturbance.

## Case presentation

Our patient was an 88-year-old Japanese man who had been undergoing eye drop treatment for angle-closure glaucoma in his left eye since 2003. In February 2008, he experienced lens luxation into the inferior vitreous cavity. He had no ocular trauma at that time, and he also had no ocular abnormalities such as pseudoexfoliation or lens subluxation. Because the lens capsule was intact and inflammation such as lens-induced endophthalmitis was not present, we observed the patient without prescribing additional medications except for the eye drop treatment for glaucoma. The patient had had phthisis bulbi of unknown origin in the fellow eye since childhood. Hence, eyeglasses were prescribed to improve his corrected VA, and he was followed conservatively. During the follow-up period, his corrected VA remained at 20/20, and intraocular pressure (IOP) was controlled to approximately 12 mmHg in his left eye. He had no systemic medical history and was taking no medication except the eye drop treatment for glaucoma. Moreover, there was nothing particular of note in regard to the patient’s family history.

**Fig. 1:**
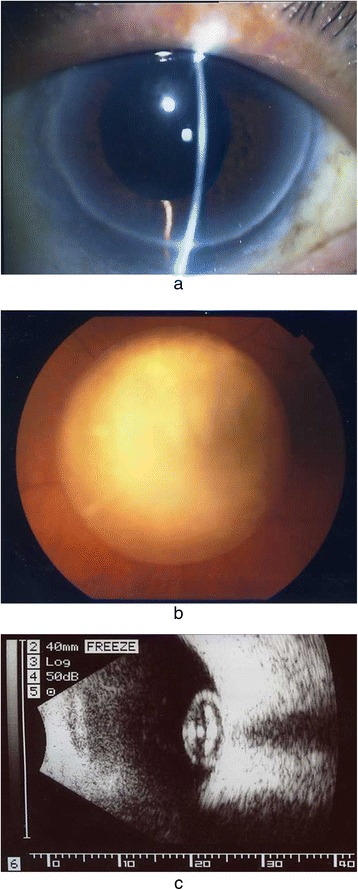
**a** Preoperative anterior segment of the patient’s left eye. Slit-lamp examination revealed absence of the crystalline lens and deep anterior chamber. **b** Preoperative fundus image of the left eye. The luxated lens in the vitreous cavity can be seen stuck to the optic disc. The patient’s visual acuity in that eye was 20/400. **c** B-mode ultrasonography findings. No movement of the luxated lens was observed in relation to changes in the patient’s head position or eye movement

In April 2010, the patient revisited our clinic after suddenly noticing a visual disturbance in his left eye. Slit-lamp examination revealed the absence of crystalline lens and deep anterior chamber (Fig. 1a). A fundus examination revealed that the patient’s luxated lens had become stuck on the optic disc and obstructed the macular region, thus causing a marked visual disturbance (Fig. 1b). The corrected VA in his left eye decreased to 20/400, but the IOP in that eye remained at 12 mmHg. B-mode ultrasonography showed no movement of the luxated lens in the vitreous cavity in relation to changes in the patient’s head position or eye movement (Fig. 1c).

**Fig. 2:**
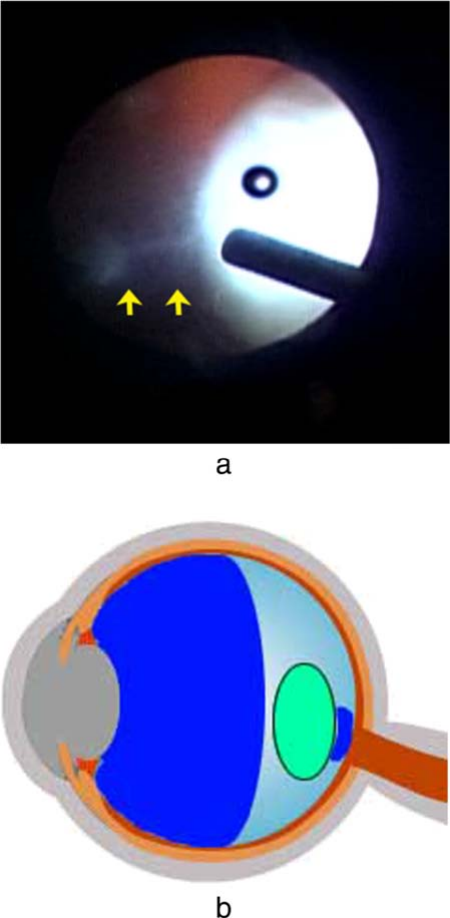
**a** Image showing the intraoperative findings. An aggregation of vitreous gel (*arrows*) on the optic disc was found. The luxated lens probably became stuck on the optic disc via this residual vitreous gel. **b** An illustration depicting this mechanism

**Fig. 3:**
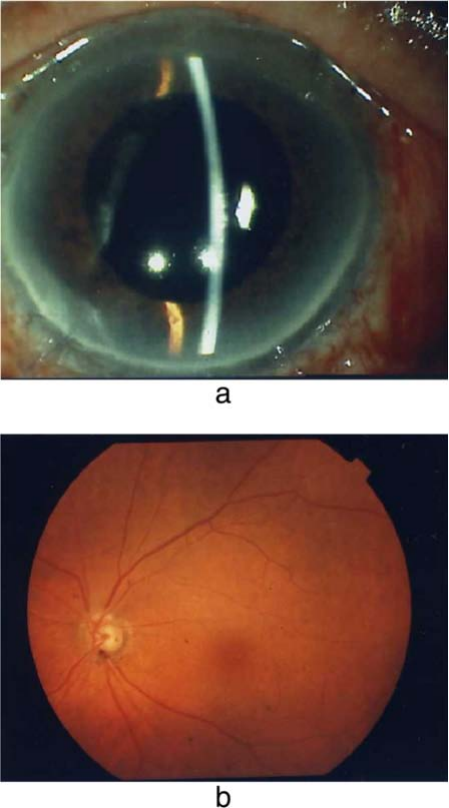
**a** Postoperative anterior segment of the patient’s left eye. Slit-lamp examination revealed no new changes. **b** Postoperative funduscopic image of the left eye. Following vitreous surgery, the macula could be visualized and the corrected visual acuity in that eye had improved to 20/25

At 7 days after the onset of decreased VA, pars plana vitrectomy was indicated to remove the luxated lens. During the surgery, posterior vitreous detachment appeared to occur. However, we observed an aggregation of vitreous gel between the luxated lens and the optic disc (Fig. 2a and b). The luxated lens was successfully mobilized by pushing it with a vitreous cutter, which eliminated the adhesion of the luxated lens to the optic disc without any difficulties or problems. The luxated lens was then floated upward using liquid perfluorocarbon and extracted through a superior 130-degree corneoscleral incision. We selected that technique on the basis of our presumption that the dislocated crystalline lens was quite hard. Following surgery the macula could be visualized, and by 4 months after surgery the corrected VA in that eye had improved to 20/25 and the IOP remained at approximately 16 mmHg without additional treatment. In addition, slit-lamp examination revealed no new changes (Fig. 3a). A funduscopic examination revealed a slightly small optic disc (disc macula to disc diameter ratio 0.3), but no congenital anomalies such as an optic disc pit or morning glory syndrome (Fig. 3b).

## Discussion

Ophthalmologists sometimes encounter patients with lens luxation into the vitreous cavity, but cases in which the luxated lens becomes stuck to the optic disc are extremely rare. In our patient, lens adhesion to the optic disc occurred approximately 2 years after complete lens luxation. The lens adhesion found in this patient may have occurred for one of the following three reasons: (1) tissue changes (such as those associated with intraocular inflammation), (2) negative pressure on the optic disc, or (3) other physical factors.

In regard to the first possibility, it has been reported that damage to the lens capsule can cause leakage of lens proteins into the vitreous cavity and elicit lens-induced endophthalmitis [7]. In such cases, cyclitic membrane formation can occur as a result of the granulomatous inflammation, and adhesions can form between the luxated lens and the optic disc. However, the gross pathological appearance of the extracted lens in our patient, other than showing cataract-related changes, exhibited no findings indicative of inflammation. A preoperative examination also revealed no inflammation of the anterior chamber and vitreous cavity. Thus, adhesion due to intraocular inflammation was ruled out.

In regard to the second possibility, if pressure in the subarachnoid space surrounding the optic nerve is decreased, negative pressure on the optic disc reportedly can occur [8, 9]. This type of negative pressure may occur in congenital anomalies of the optic disc, such as optic disc pits and morning glory syndrome. In such cases, migration of silicone oil from the vitreous cavity into the brain and migration of air and silicone oil in the vitreous cavity into the subretinal space have occasionally been reported [10–12]. However, although our patient had a small optic disc, no congenital anomalies were observed. Thus, adhesion of the luxated lens due to negative pressure on the optic disc was deemed unlikely.

In regard to the third possibility, physical factors such as residual vitreous cortex were associated with the pathological condition in our patient. Intraoperative findings revealed the occurrence of posterior vitreous detachment; however, we observed an aggregation of vitreous gel between the luxated lens and optic disc. The adhesion between the optic disc and vitreous is anatomically strong, so even if posterior vitreous detachment occurs, some remnant vitreous gel may be present. In fact, this aggregation of vitreous gel on the optic disc appeared to play the role of an interpositional material between the luxated lens and the optic disc in our patient. Fetal vitreous tissue, such as the Cloquet’s canal, is reportedly occasionally seen in congenital anomalies of the optic disc [13–15]. In our patient, no abnormality of the optic disc was found, but the possibility that a similar tissue was present cannot be ruled out.

## Conclusions

Our findings in this case suggest that the luxated lens had become stuck on the optic disc via residual vitreous gel on the disc.

## Consent

Written informed consent was obtained from the patient for publication of this case report and any accompanying images. A copy of the written consent is available for review by the Editor-in-Chief of this journal.

## Abbreviations

IOP: intraocular pressure
VA: visual acuity
